# METTL3 Promotes Cutaneous T-Cell Lymphoma Progression by Regulating ARHGEF12 Expression

**DOI:** 10.3390/ijms26083640

**Published:** 2025-04-11

**Authors:** Lu Gan, Yingqi Kong, Haoze Shi, Congcong Zhang, Cuicui Tian, Hao Chen

**Affiliations:** Hospital for Skin Diseases, Institute of Dermatology, Chinese Academy of Medical Sciences and Peking Union Medical College, Nanjing 210042, China; gl90gl@pumcderm.cams.cn (L.G.); airswo@163.com (Y.K.); shihaoze@aliyun.com (H.S.); zccjy0614@163.com (C.Z.); tianccsysu@163.com (C.T.)

**Keywords:** METTL3, m6A, ARHGEF12

## Abstract

Recent studies have identified N6-methyladenosine (m6A) RNA methylation as a key regulatory mechanism in tumor progression. This study aimed to elucidate the biological function and clinical relevance of the m6A methyltransferase METTL3 in cutaneous T-cell lymphoma (CTCL). Our findings demonstrated that METTL3 expression is upregulated in CTCL, and its knockdown suppresses CTCL progression. Mechanistically, the downregulation of METTL3-mediated m6A modification on ARHGEF12 mRNA accelerated its degradation, a process that is closely associated with tumor behaviors. These results suggest that METTL3 may serve as a potential therapeutic target in CTCL.

## 1. Introduction

Cutaneous T-cell lymphoma (CTCL) represents a subgroup of extranodal non-Hodgkin lymphomas, characterized by clonal malignant T-lymphocytes that proliferate and infiltrate the skin [1]. The incidence of CTCL is estimated at approximately 6 to 10 cases per million individuals. The majority of CTCLs are classified as mycosis fungoides (MF), Sézary syndrome, and primary cutaneous anaplastic large-cell lymphoma (pcALCL), with MF being the most prevalent, comprising over 60% of all CTCL cases [2,3].

The management of cutaneous T-cell lymphoma (CTCL) is generally stratified according to disease stage. Skin-directed therapies are the primary approach for patch or plaque-stage disease with limited skin involvement. Common therapeutic agents include topical corticosteroids, topical bexarotene, and topical mechlorethamine hydrochloride. Additionally, localized radiation therapy, including psoralen plus ultraviolet A (PUVA), narrowband ultraviolet B (NB-UVB), and electron beam therapy, is highly effective with minimal toxic effects. Advanced-stage CTCL is characterized by its aggressive clinical course and resistance to conventional therapies [4]. Significant advancements have been achieved over the past five years, particularly with the approval of histone deacetylase inhibitors (HDACi), brentuximab vedotin (BV), and mogamulizumab. Pembrolizumab has demonstrated efficacy, while novel agents such as E7777 and lacutamab are currently under investigation [5].

Histone deacetylases (HDACs) are ubiquitously expressed enzymes that catalyze the removal of acetyl groups from histones, leading to chromatin condensation and transcriptional repression. HDAC inhibitors (HDACis) induce tumor cell cycle arrest and apoptosis by inhibiting specific HDAC isoforms, thereby increasing histone acetylation and promoting chromatin remodeling. Representative HDACis include cedarbenamide, vorinostat, and romidepsin. A multicenter prospective trial reported an overall response rate of 39.06% for cedarbenamide monotherapy in refractory peripheral T-cell lymphoma, which increased to 51.18% when combined with chemotherapy [6]. Brentuximab vedotin (BV), an anti-CD30 antibody–drug conjugate, has been approved by the U.S. Food and Drug Administration (FDA) and the European Medicines Agency (EMA) for the treatment of CD30-positive mycosis fungoides (MF). BV is also indicated for relapsed or refractory classical Hodgkin lymphoma. CD30 expression is widely observed in lymphomatoid papulosis (LyP) and primary cutaneous anaplastic large-cell lymphoma (pcALCL). In MF and Sézary syndrome (SS), the proportion of CD30-positive tumor cells ranges from approximately 12% to 23%, increasing to 48% to 55% following large-cell transformation in MF patients. In an 18-month follow-up study conducted by Muniesa involving 67 patients (48 with MF, 7 with SS, and 12 with CD30+ lymphoproliferative disorders [LPD]), BV achieved an overall response rate (ORR) of 67% (63% in MF, 71% in SS, and 84% in CD30+ LPD). The median time to response was 2.8 months. During follow-up, 54% of patients (*n* = 36) experienced disease recurrence or progression in the skin, with a median progression-free survival (PFS) of 10.3 months [7]. Mogamulizumab, a humanized monoclonal antibody targeting CC chemokine receptor 4 (CCR4), was evaluated in a Japanese cohort of 28 patients with CTCL, demonstrating an ORR of 39.3% [8]. A phase III trial comparing mogamulizumab with vorinostat in refractory MF/SS reported a median PFS of 7.7 months with mogamulizumab, significantly longer than the 3.1 months observed in the vorinostat group [9].

The emerging therapeutic agents currently under development primarily serve to mitigate disease progression rather than achieving a sustained therapeutic response in patients. This highlights the urgent need to elucidate the molecular mechanisms driving CTCL progression to identify potential therapeutic targets.

Increasing evidence underscores the complexity of CTCL pathogenesis, involving diverse alterations at epigenetic, genetic, and proteomic levels [10,11,12]. N6-methyladenosine (m6A) is the most abundant internal mRNA modification in eukaryotic cells, playing a critical role in regulating mRNA stability and translation. This modification is dynamically modulated by methyltransferases (“writers”), demethylases (“erasers”), and m6A-binding proteins (“readers”). METTL3, an m6A “writer”, is a key methyltransferase involved in promoting cell proliferation, migration, and various other biological processes [13]. Recent advances in m6A research have revealed its role in tumor progression and drug resistance in numerous cancers, including hepatocellular carcinoma, lung cancer, and gastric cancer [14,15,16,17]. Elevated METTL3/METTL14 levels have been shown to drive the growth of several cancer types, and METTL3/METTL14 inhibitors have emerged as potential therapeutic agents [18].

Interestingly, only one study has reported that the METTL3-CDKN2A axis inhibits CTCL cell proliferation and migration [19]. The precise role of m6A dysregulation in CTCL pathogenesis remains unclear. In this study, we investigated the mechanisms underlying m6A-mediated METTL3 dysregulation in CTCL, revealing that METTL3 modulates ARHGEF12 expression. Our findings indicate that METTL3 may serve as a novel prognostic biomarker and therapeutic target in CTCL, providing insights into m6A-driven oncogenic processes in this challenging malignancy.

## 2. Results

### 2.1. Elevated METTL3 Expression in MF Tissues and CTCL Cell Lines

**Figure 1:**
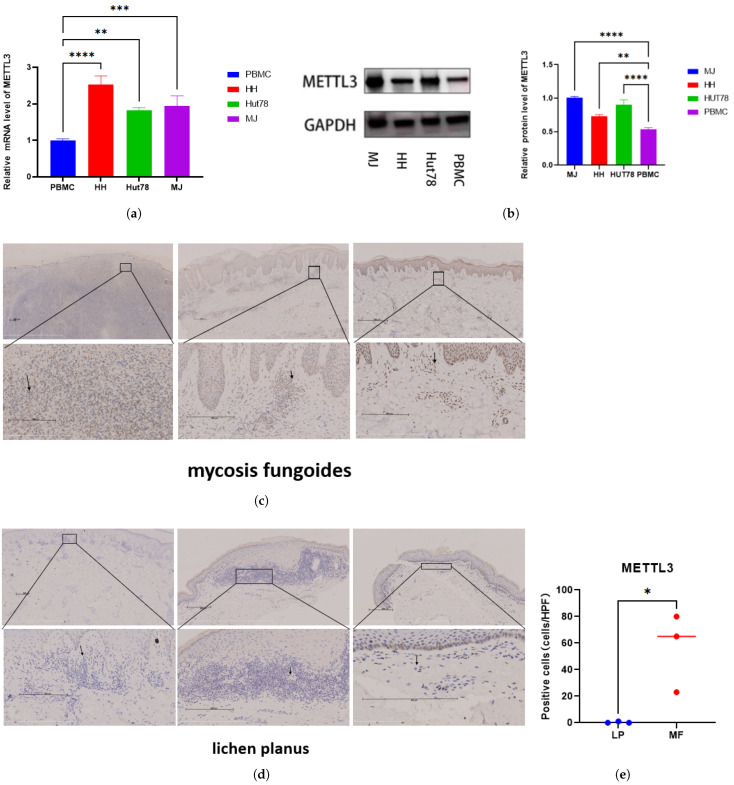
METTL3 expression was elevated in CTCL: METTL3 level in PBMC and cutaneous T-cell lymphoma cell lines (HH, Hut78, and MJ) verified by (**a**) RT-qPCR and (**b**) Western blot. The values are represented in technical replicates. The METTL3 protein level in (**c**) mycosis fungoides tissues and (**d**) lichen planus tissues assessed by immunohistochemistry. Arrows point to lymphoma cells in MF and lymphocytes in LP. (**e**) METTL3-positive cells in the dermis were counted per high power field (×400). Scale bar = 200 μ m. Data are shown as means ± S.D. and statistical significance is denoted as * *p* < 0.05, ** *p* < 0.01, *** *p* < 0.001, and **** *p* < 0.0001.

We performed RT-qPCR and Western blot to detect mRNA and protein levels of METTL3 in three CTCL cell lines (HH, MJ, and Hut78) and peripheral blood mononuclear cells of healthy donors. We found that mRNA (Figure 1a) and protein levels (Figure 1b) of METTL3 were highly expressed in tumor cells compared with healthy donors. Immunohistochemical results revealed that METTL3 was highly expressed in MF tissue samples (Figure 1c) compared with lichen planus (Figure 1d). The number of METTL3-positive cells in the dermis in MF skin was significantly higher than that of normal skin (Figure 1e). These findings suggested that METTL3 was highly expressed in MF tissues and CTCL cell lines and may have an impact on malignant behavior.

### 2.2. Knockdown METTL3 Suppresses CTCL Progression

**Figure 2:**
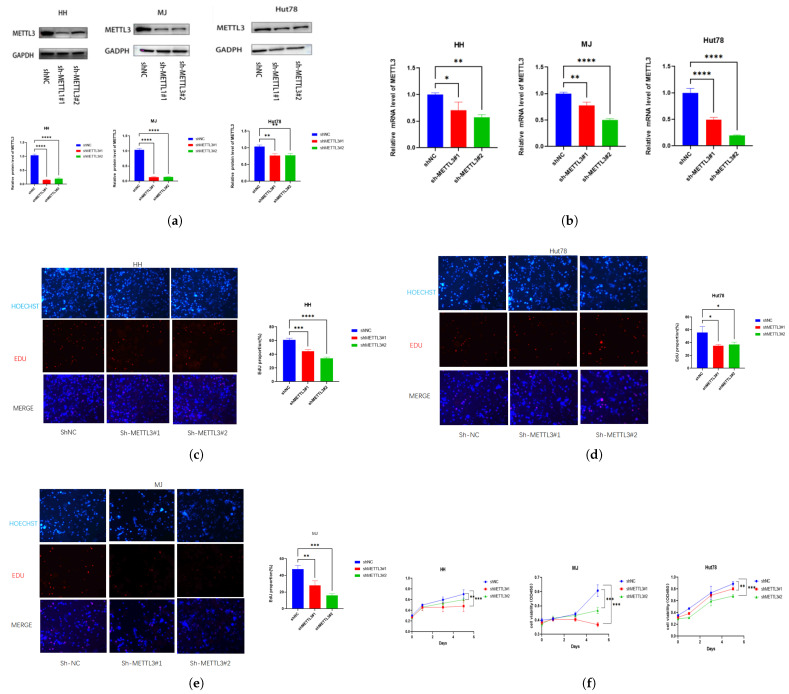
METTL3 downregulation inhibits CTCL cell proliferation: The efficacy of METTL3 knockdown was confirmed by (**a**) Western blot and (**b**) RT-qPCR. (**c**–**e**) Cell proliferation assays (EdU) and (**f**) CCK8 revealed that METTL3 downregulation markedly inhibited the proliferation capacity of HH, MJ, and Hut78 cells. All data are presented as mean ± S.D. and statistical significance is denoted as * *p* < 0.05, ** *p* < 0.01, *** *p* < 0.001, and **** *p* < 0.0001.

**Figure 3:**
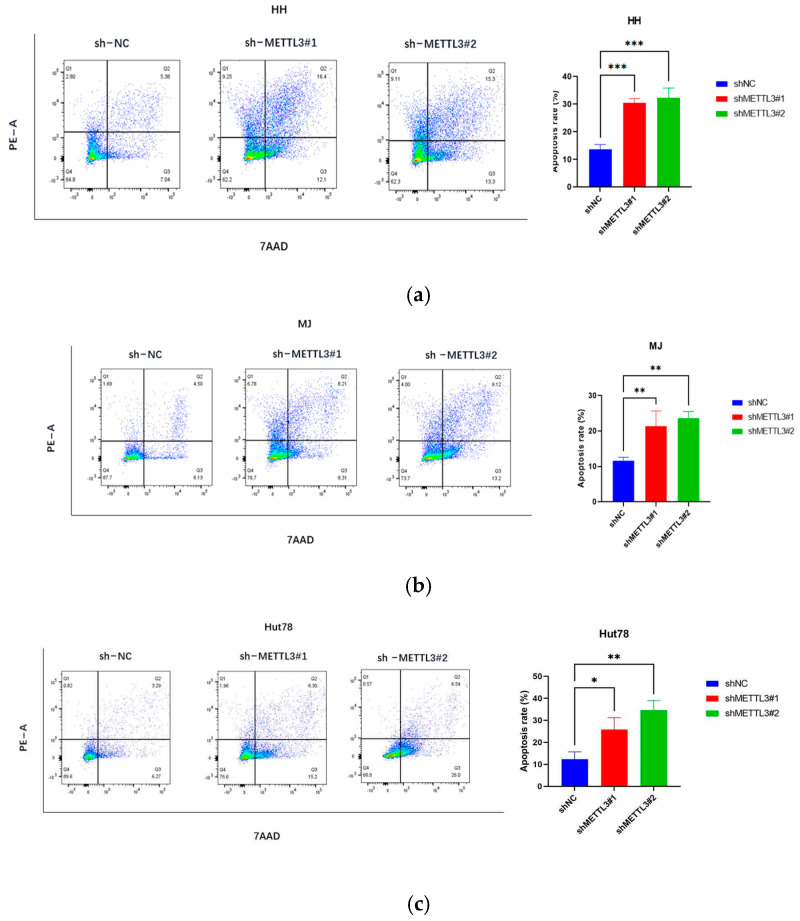

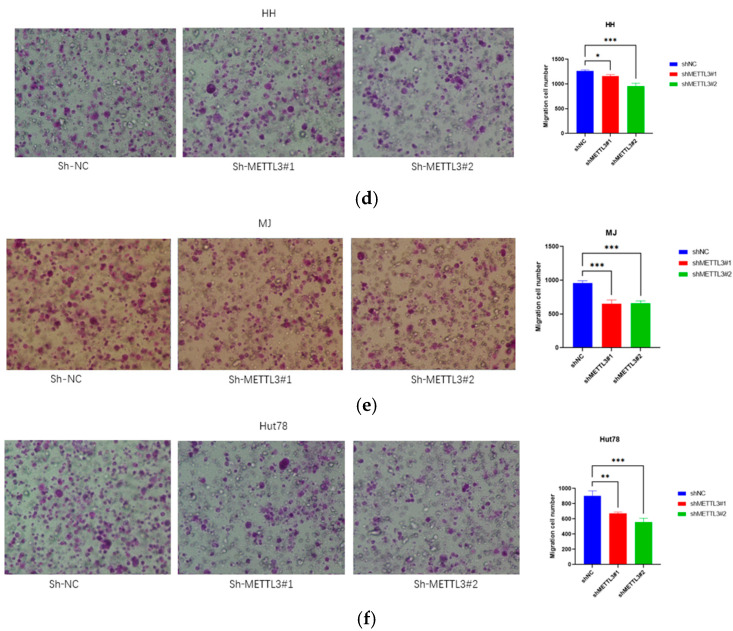
METTL3 downregulation inhibits CTCL cell invasion and promotes apoptosis: (**a**–**c**) Apoptosis assays showed significantly increased apoptosis rates in CTCL cells following METTL3 knockdown. (**d**–**f**) Transwell experiments indicated that METTL3 downregulation significantly impaired the migration ability of HH, MJ, and Hut78 cells within 16 h. All data are presented as mean ± S.D. and statistical significance is denoted as * *p* < 0.05, ** *p* < 0.01, and *** *p* < 0.001.

To assess METTL3’s role in CTCL, stable METTL3-knockdown cells were generated, with knockdown efficacy validated via Western blotting (Figure 2a) and qPCR (Figure 2b). Knockdown of METTL3 significantly reduced proliferation measured by EdU (Figure 2c–e) and CCK8 (Figure 2f) assays. METTL3 knockdown also significantly increased CTCL cell apoptosis, which was measured by the 7AAD/PE Annexin V assay (Figure 3a–c). The migration assay showed that knockdown METTL3 significantly inhibited the ability of migration in CTCL cells (Figure 3d–f).

### 2.3. The Downstream Targets of METTL3 Identified by Multi-Omics Analysis

**Figure 4:**
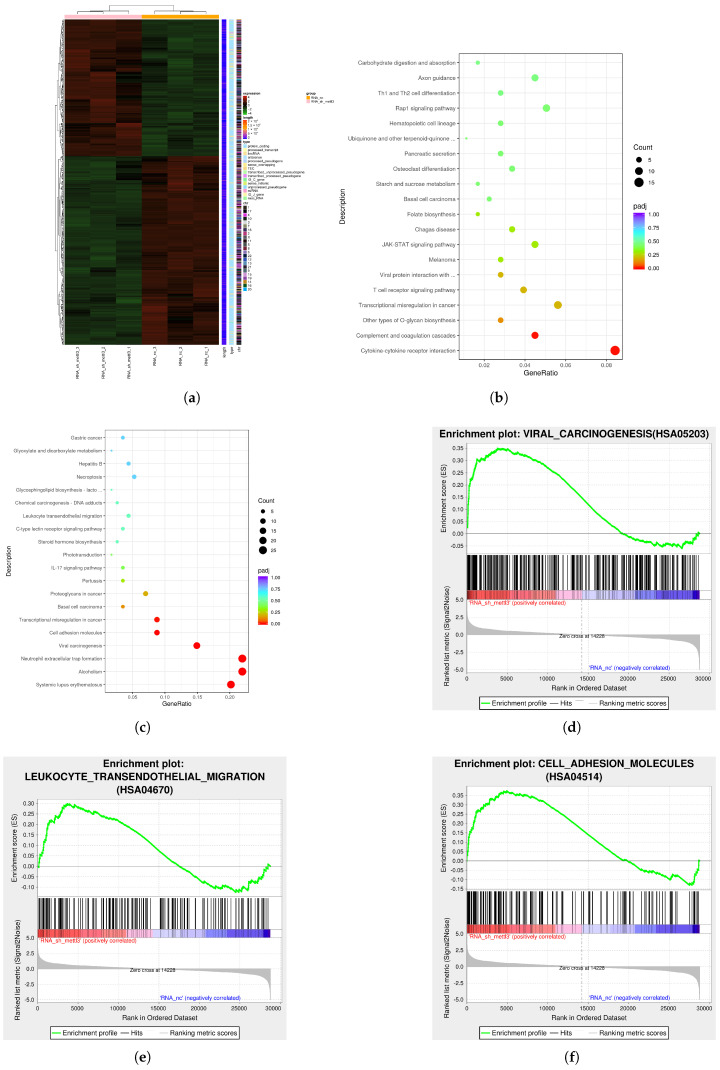
Transcript profile of RNA−seq regulated by METTL3 in HH cell: (**a**) Heatmap of differentially expressed genes (DEGs) identified by RNA-seq between shNC group and sh−METTL3 group. KEGG enrichment analysis of (**b**) downregulated and (**c**) upregulated DEGs. (**d**–**f**) Pathways of DEGs revealed by GSEA analysis.

**Figure 5:**
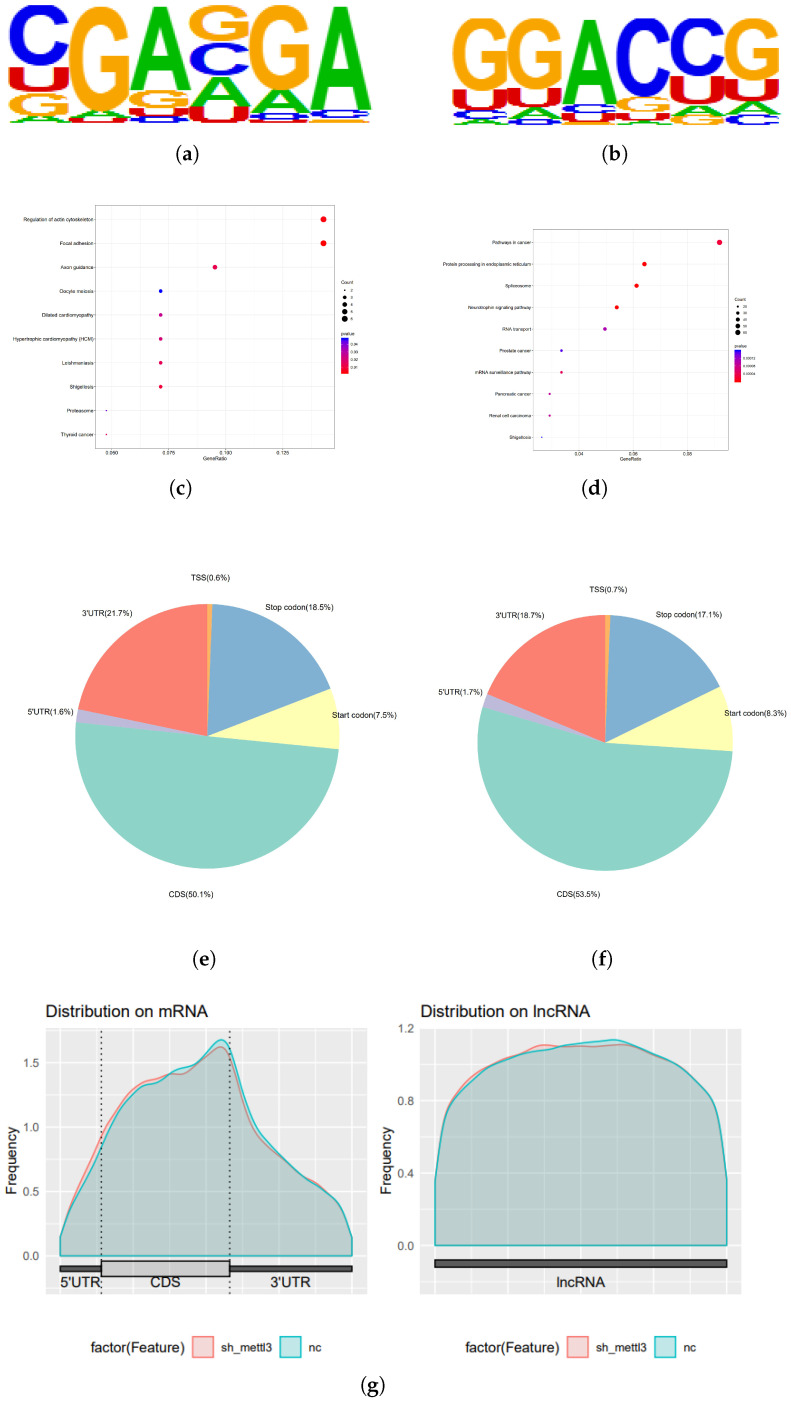
Transcript profile of MeRIP-seq regulated by METTL3 in HH cells: (**a**,**b**) The m6A motif detected by the MEME motif analysis. KEGG enrichment analysis of genes with differentially (**c**) downregulated and (**d**) upregulated expressed m6A levels in MeRIP-seq between shNC group and shMETTL3 group. (**e**,**f**) Pie diagram of m6A peak distribution on RNA structure in MeRIP-seq between shNC group and the shMETTL3 group. (**g**) Metagene plot of m6A peak distribution on structures of mRNA and lncRNA in MeRIP-seq between shNC group and shMETTL3 group.

To identify the targets regulated by METTL3, RNA-seq and MeRIP-seq were performed in HH-NC and HH-shMETTL3#1. RNA-seq analysis revealed that after METTL3 downregulation, 717 genes were significantly altered (*p* < 0.05 and |log2FoldChange| ≥ 1) (Figure 4a). Among these, 416 genes were downregulated while 301 genes were upregulated. KEGG pathway analysis of the RNA-seq data indicated significant enrichment of genes associated with transcriptional misregulation in cancer, proteoglycans in cancer, cytokine–cytokine receptor interactions, viral carcinogenesis, and cell adhesion (Figure 4b,c). Gene Set Enrichment Analysis (GSEA) further revealed that the differentially expressed genes were linked to viral carcinogenesis (such as HIST1H2BJ, HIST1H4D, HIST1H4I, EIF2AK2, HIST1H2BK, and so on), leukocyte transendothelial migration (such as ITGAM, CLDN14, GNAI1, MYL5, PIK3R2, and so on), and cell adhesion molecules (such as CLDN, JAM1, JAM2, JAM3, OCLN, and so on) (Figure 4d–f). These findings suggest that METTL3 plays a crucial role in regulating the proliferation and migration of CTCL cells. MeRIP-seq analysis of shNC and shMETTL3#1 HH cells identified 33,901 m6A peaks across 13,925 transcripts, with the m6A consensus motif GGAC being present in both groups (Figure 5a,b), indicating substantial enrichment of m6A-modified RNAs. KEGG analysis of MeRIP-seq data revealed that the enriched genes were involved in RNA transport, cell cycle regulation, RNA splicing, mRNA processing, and cancer-related pathways (Figure 5c,d). Furthermore, m6A modifications were predominantly found in the CDS and $3^{\prime }$ UTR regions (Figure 5e–g).

### 2.4. METTL3 Reduces ARHGEF12 mRNA Stability

**Figure 6:**
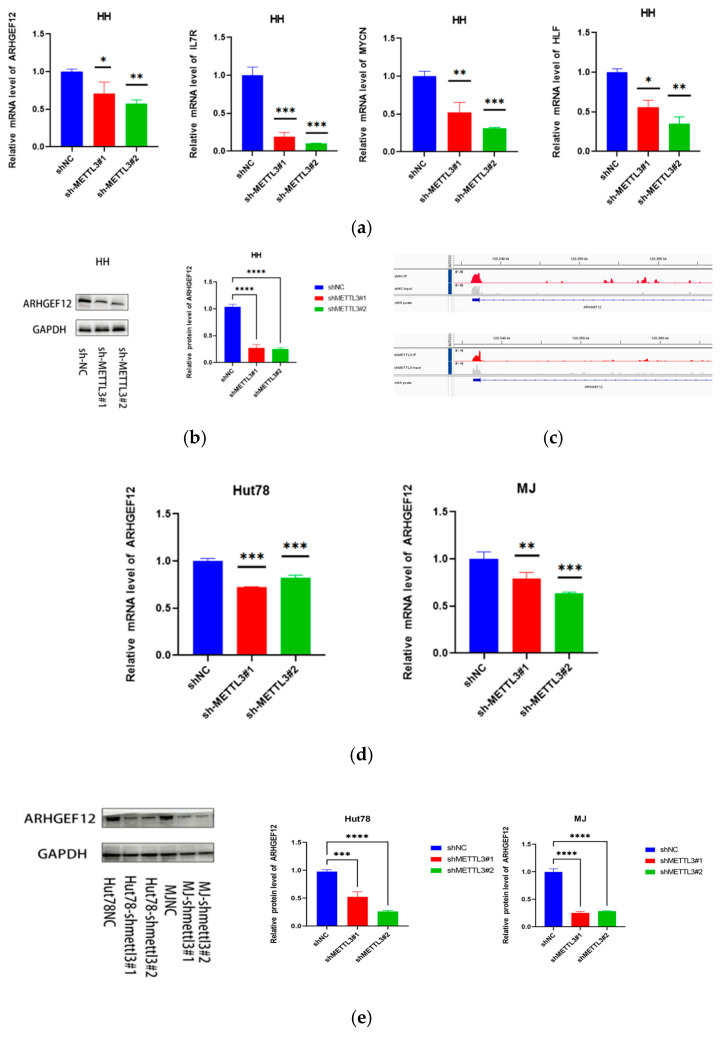

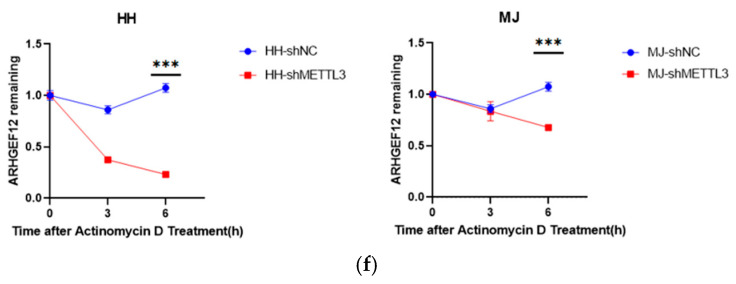
METTL3 affected ARHGEF12 mRNA stability: (**a**) MYCN, IL7R, ARHGEF12, and HLF mRNA levels in shNC and shMETTL3 cells in HH. (**b**) ARHGEF12 protein level in shNC and shMETTL3 cells in HH cells. (**c**) M6A peak in the $3^{\prime }$ UTR of ARHGEF12 in both shNC and shMETTL3 HH cells. (**d**,**e**) ARHGEF12 mRNA and protein levels in shNC and shMETTL3 cells in MJ and Hut78 cell lines. (**f**) RT-qPCR analysis of ARHGEF12 after actinomycin D treatment in shMETTL3 or control HH and MJ cells. All data are presented as the mean ± S.D. and statistical significance is denoted as * *p* < 0.05, ** *p* < 0.01, *** *p* < 0.001, and **** *p* < 0.0001.

Analysis of transcripts enriched with m6A sites bound by METTL3, as identified by MeRIP-seq, focused on overlapping targets with those found in RNA-seq data, four key transcripts were identified: MYCN, IL7R, ARHGEF12, and HLF (Figure 6a). Among these, ARHGEF12 was notably one of the most downregulated genes following METTL3 knockdown. ARHGEF12 has previously been reported as a proto-oncogene in several tumor types [20,21,22]. The protein level of ARHGEF12 also significantly decreased following METTL3 knockdown in the HH cell line (Figure 6b). Moreover, the m6A peak in the $3^{\prime }$ UTR of ARHGEF12 was prominent in both shNC and shMETTL3 HH cells (Figure 6c). Subsequent analysis of ARHGEF12 mRNA (Figure 6d) and protein (Figure 6e) expression in other CTCL cell lines, including Hut78 and MJ, revealed that ARHGEF12 expression was significantly lower in METTL3 knockdown cells compared to control cells. Given that METTL3 is generally considered an oncogene that catalyzes m6A modification of target genes, we treated MJ-shMETTL3#1 and HH-shMETTL3#1 cells with a transcription inhibitor (Actinomycin D) for various durations. This treatment resulted in a quicker rate of mRNA decay in METTL3 knockdown cells, indicating that METTL3 regulates the stability of ARHGEF12 mRNA (Figure 6f).

### 2.5. Elevated ARHGEF12 Expression in CTCL and MF Patients

**Figure 7:**
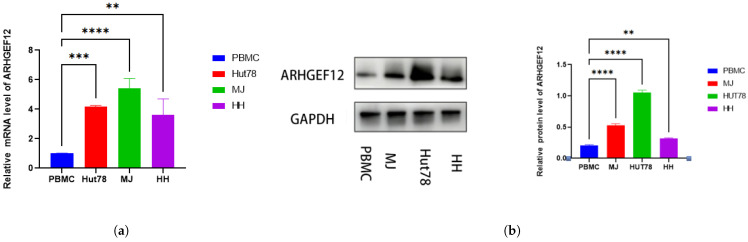

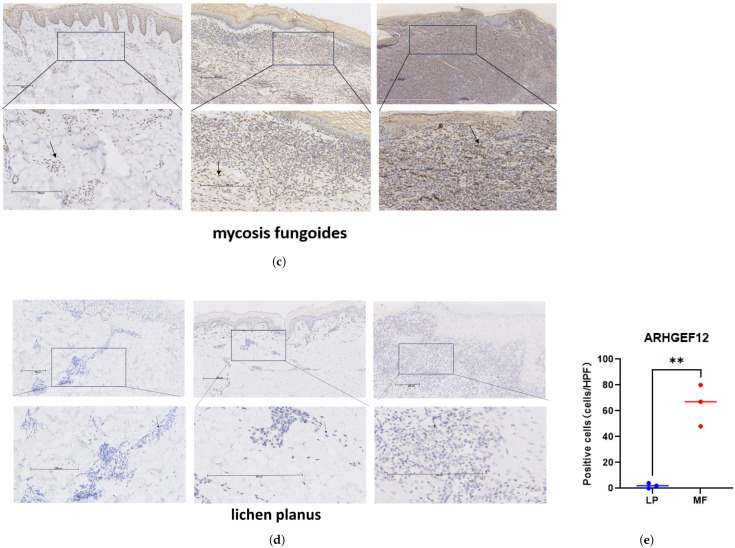
ARHGEF12 expression was elevated in CTCL: ARHGEF12 level in PBMC and cutaneous T-cell lymphoma cell lines (HH, Hut78, and MJ) verified by (**a**) RT-qPCR and (**b**) Western blot. The ARHGEF12 protein level in (**c**) mycosis fungoides tissues and (**d**) lichen planus tissues tissues assessed by immunohistochemistry. Arrows point to lymphoma cells in MF and lymphocytes in LP. (**e**) ARHGEF12-positive cells in the dermis were counted per high power field (×400). Scale bar = 200 μ m. Data are shown as means ± S.D. and statistical significance is denoted as ** *p* < 0.01, *** *p* < 0.001, and **** *p* < 0.0001.

We then performed RT-qPCR and Western blot to compare mRNA and protein levels of ARHGEF12 in three CTCL cell lines (HH, MJ, and Hut78) and peripheral blood mononuclear cells of healthy donors. We found that mRNA (Figure 7a) and protein levels (Figure 7b) of ARHGEF12 were highly expressed in tumor cells. Immunohistochemical results revealed that ARHGEF12 was highly expressed in MF tissue samples (Figure 7c) compared with lichen planus tissues (Figure 7d). The number of ARHGEF12-positive cells in the dermis in MF skin was significantly higher than that of normal skin (Figure 7e). These findings suggested that ARHGEF12 was highly expressed in MF tissues and cell lines.

### 2.6. METTL3 Promotes Tumor Growth In Vivo

**Figure 8:**
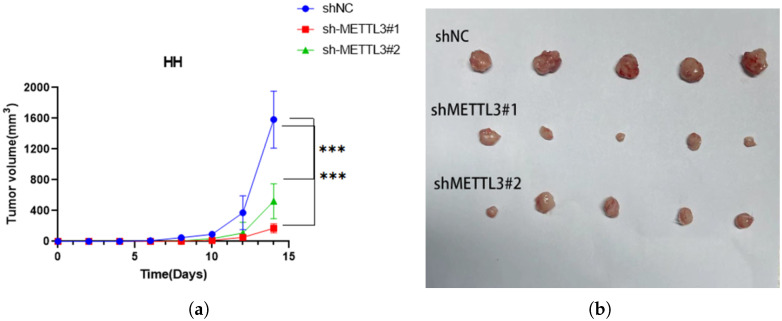

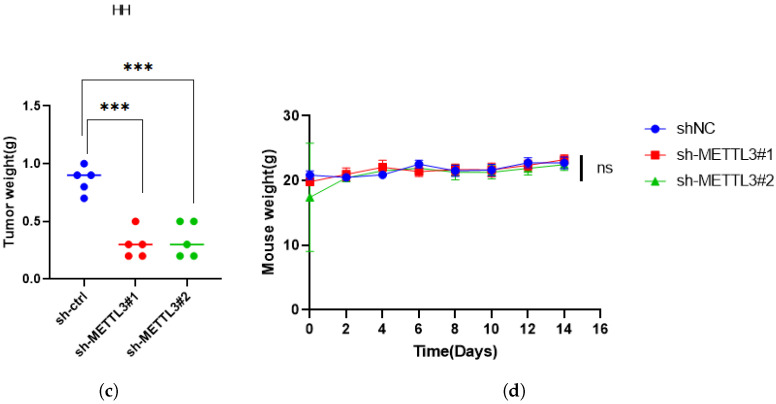
METTL3 promotes tumor growth in vivo: HH cells with or without METTL3 deficiency in 200 μ L of PBS were injected into the shaved right flank of NSG mice. On days 0, 2, 4, 6, 8, 10, 12, and 14, tumor volume was calculated as (length × ${\mathrm{width}}^{2}$ )/2. The mice were sacrificed 14 days post-injection. (**a**) Tumor growth curves after the injection of shMETTL3 and control HH cells. (**b**) Xenograft tumors in each group were shown. (**c**) Tumor weight was calculated as well. (**d**) The weights of mice were monitored every other day and showed no significant difference. Data are shown as means ± S.D. *** means *p* < 0.001.

**Figure 9:**
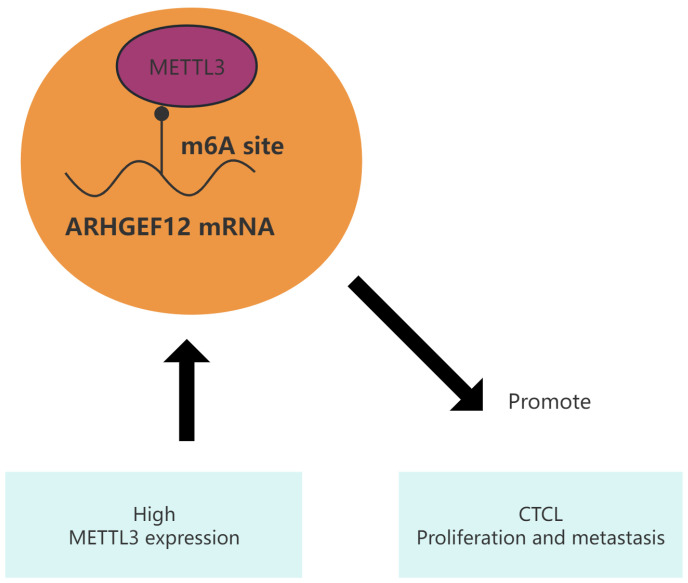
Schematic diagram of METTL3 regulating its “executor” ARHGEF12 in CTCL.

In order to explore the role of METTL3 in regulating tumorigenesis in vivo, HH cells with or without METTL3 knockdown were injected into NSG nude mice. We found that METTL3 knockdown efficiently delayed tumor growth (Figure 8a–c) while the weight of mice showed no significant difference (Figure 8d). All of above results confirmed the oncogenic role of METTL3 in CTCL by promoting cell proliferation in vivo (Figure 9). Collectively, these results demonstrated that METTL3 recognizes the m6A sites on ARHGEF12 and promotes its protein expression by affecting its mRNA stability.

## 3. Discussion

N6-methyladenosine (m6A) is the most prevalent post-transcriptional modification of messenger RNA (mRNA) in eukaryotic cells. It exerts profound effects on RNA processing, translation, and degradation, thereby influencing cellular differentiation and tissue development. Aberrations in the regulation of m6A have been implicated in numerous human diseases, including cardiovascular disorders, metabolic syndromes, viral infections, and cancer progression [23,24,25]. M6A modifications are dynamically regulated by “writers” (methyltransferases), “erasers” (demethylases), and “readers” (methylation recognition proteins). Dysregulation of this modification has been observed across diverse malignancies, where it facilitates cancer progression by modulating mRNA stability, translation, and splicing [26].

Previous studies have suggested that METTL3, a key m6A methyltransferase, inhibits the proliferation and migration of cutaneous T-cell lymphoma (CTCL) cells by regulating the expression of CDKN2A [19]. However, our experimental findings reveal contradictory results, suggesting a potential oncogenic role of METTL3 in CTCL.

CTCLs represent a heterogeneous group of T-cell lymphomas primarily confined to the skin, with no evidence of extracutaneous involvement at diagnosis. These subtypes exhibit diverse clinical and molecular characteristics, ranging from indolent to highly aggressive courses. Among these, mycosis fungoides (MF) is the most common subtype, accounting for over 60% of CTCL cases [27]. Current therapeutic strategies for advanced MF and Sézary syndrome (SS) remain limited [28]. While systemic chemotherapy offers high initial response rates, the responses are typically transient and associated with substantial toxicities [29]. Consequently, there is a pressing need for novel therapeutic targets for these challenging malignancies, yet the underlying disease biology remains poorly understood due to its rarity [30].

Emerging evidence highlights the role of m6A-associated proteins in lymphoma progression. For instance, METTL3 is significantly upregulated in natural killer/T-cell lymphoma (NKTCL), where it drives tumorigenesis through the METTL3/EBV-miR-BART3-3p/PLCG2 axis [31]. Similarly, YTHDF2 promotes diffuse large B-cell lymphoma (DLBCL) progression by modulating ACER2-mediated ceramide metabolism in an m6A-dependent manner [32]. In T-cell acute lymphoblastic leukemia (T-ALL), IGF2BP2 binds to the oncogene NOTCH1 via an m6A-dependent mechanism and contributes to leukemogenesis [33]. Moreover, inhibition of FTO, an m6A demethylase, significantly suppresses leukemic cell proliferation and prolongs survival in T-ALL models by restoring IRF8 expression [34]. Furthermore, mortalin gained increased mRNA stability and enhanced translation efficiency via the m6A methylation in the HSPA9 mRNA 3’UTR, which was catalysed by METTL3 in cervical cancer cells [35]. Notably, the oncogenic role of METTL3 often depends on its interaction with METTL14, suggesting that protein–protein interaction (PPI)-based therapeutic strategies may hold promise.

In the context of CTCL, current research suggests a tumor-suppressive role for METTL3 by modulating CDKN2A expression [19]. However, the evidence remains inconclusive regarding whether METTL3 inhibition might have broader therapeutic implications. To address this gap, we investigated the role of METTL3 in CTCL pathogenesis and its underlying molecular mechanisms.

Our study shows that METTL3 expression is increased in mycosis fungoides compared with the inflammatory disease lichen planus, revealing that elevated METTL3 expression may act as an oncogenic driver in CTCL progression. Mechanistically, METTL3 recognizes m6A-modified sites on ARHGEF12, thereby enhancing its protein expression. The METTL3/ARHGEF12 axis plays a pivotal role in CTCL progression and represents a potential predictive biomarker and therapeutic target for this disease. While the findings of this study provide valuable insights into the new target for CTCL, it is important to recognize that the relatively small number of cases included in this analysis is a limitation, which highlights the need for continued investigation in this area.

## 4. Materials and Methods

### 4.1. Tissue Sample Collection

Six tissue samples, including three cases of mycosis fungoides (MF) and three cases of lichen planus, were fixed in formalin and embedded in paraffin. All samples were acquired from the Institute of Dermatology, Chinese Academy of Medical Sciences. Informed consent was obtained from each patient for this study (2023-KY-048).

### 4.2. Cell Culture

The human CTCL cell line Hut78 was purchased from CoBioer Biotechnology (Nanjing, China). HH and MJ cell lines were generously provided by Professor Yang Wang at Peking University First Hospital. HH and Hut78 cells were maintained in RPMI-1640 medium supplemented with 10% fetal bovine serum (FBS), 100 U/mL penicillin, and 100 μ g/mL streptomycin. MJ cells were cultured in IMDM medium with 20% FBS and 1% penicillin and streptomycin. All cells were incubated at 37 °C with 5% CO_2_ in a BB150 incubator (Thermo Fisher Scientific Inc., Langenselbold, Germany).

### 4.3. RNA Isolation and RT-qPCR

Total RNA was extracted from cells using RNAiso Plus (Takara, Dalian, China) following the manufacturer’s instructions. The extracted RNA was reverse transcribed into cDNA using the PrimeScript™ RT Master Mix (Accurate Biology, Changsha, China). Samples were analyzed on a Roche LC480 system, with expression normalized to β -actin. The primers used in this study were as follows: β -actin (F: $5^{\prime }$ GTGGCCGAGGACTTTGATTG $3^{\prime }$ , R: $5^{\prime }$ CCTGTAACAACGCATCTCATATT $3^{\prime }$ ); ARHGEF12 (F: $5^{\prime }$ ACACAGTCTACTATCACCGACA $3^{\prime }$ , R: $5^{\prime }$ TGCAATGCGCTCAACTTTCTG $3^{\prime }$ ); and METTL3 (F: $5^{\prime }$ TTGTCTCCAACCTTCCGTAGT $3^{\prime }$ , R: $5^{\prime }$ CCAGATCAGAGAGGTGGTGTAG $3^{\prime }$ ).

### 4.4. Western Blotting

Cells were lysed in RIPA buffer with protease and phosphatase inhibitor cocktails. Protein concentrations were quantified using an enhanced BCA assay. Samples (20 μ g total protein) were separated via SDS-PAGE and transferred onto 0.45 μ m PVDF membranes. Membranes were blocked with 5% BSA for 1 h and incubated overnight at 4 °C with primary antibodies (anti-METTL3, 1:1000, Proteintech, Wuhan, China; anti-ARHGEF12, 1:1000, Proteintech, Wuhan, China). After washing with TBST, membranes were incubated with secondary antibodies at room temperature for 1.5 h.

### 4.5. Immunohistochemistry (IHC)

Paraffin-embedded tissue specimens were sectioned, deparaffinized in xylene and rehydrated. Antigenic retrieval was processed with sodium citrate. The sections were then incubated in H_2_O_2_ (3%) for 10 min, blocked in 1% bovine serum albumin for 20 min and incubated with anti-METTL3 (1:800, Proteintech) and anti-ARHGEF12 (1:200, Proteintech) at 4 °C overnight. After incubation with the secondary antibody for 20 min, specimens were incubated with H_2_O_2_-diaminobenzidine until the desired stain intensity was developed. Sections were then counterstained with haematoxylin, dehydrated, and mounted. Staining was analyzed by Ying Zhang, Haoze Shi, and Hao Chen from our institution.

### 4.6. Plasmid Construction and Lentiviral Transfection

To construct the lentiviral vector carrying shRNA targeting METTL3, complementary sense and antisense oligonucleotides were synthesized and subsequently cloned into the GV112 vector (hU6-MCS-CMV-puromycin). The sequence of sgNC was TTCTCCGAAC-GTGTCACGT. The shMETTL3 sequences designed to target METTL3 were as follows: ACCCACCTCTGGTGGCCCTAA; GTGCAGAACAGGACTCGACTA; and AGGCTCAACATACCCGTACTA. All lentiviral vectors and plasmids were generated by GeneChem (Shanghai, China). HH, Hut78, and MJ cells were transduced with lentiviral supernatants carrying shMETTL3 and were selected with 2 μ g/mL puromycin for 48 h post-transfection.

### 4.7. CCK8 Assay

The proliferation of tumor cells was assessed using the CCK8 assay. Hut78 and HH cells were seeded into 96-well plates at a density of 8000 cells per well, and MJ cells at 4000 cells per well. Cells were cultured for 0, 24, 72, and 120 h, followed by the addition of 10 μ L of CCK8 solution. Absorbance at 450 nm was measured after 4 h using a Multiskan Spectrum (Thermo Scientific, Waltham, MA, USA).

### 4.8. EdU Cell Proliferation Assay

Proliferation was further quantified using the BeyoClick™ EdU Cell Proliferation Kit (Beyotime Biotechnology, Shanghai, China). HH, Hut78, and MJ cells were first labeled with EdU, followed by fixation, washing, permeability, and staining. The absorbance was measured at 370 nm after coloring the sample.

### 4.9. Apoptosis Detection

CTCL cells were seeded in 6-well plates at a density of 300,000 cells per well and cultured for 48 h. After washing with cold PBS, cells were suspended in 500 μ L binding buffer, stained with 5 μ L 7-AAD and 5 μ L PE Annexin V (BD, Apoptosis Detection Kit I, 559763), and analyzed by BD FACS Calibur flow cytometry.

### 4.10. Transwell Experiment

HH, MJ, and Hut78 cells were conducted in a 24-well transwell chamber system (Corning Inc., Corning, NY, USA). A total of 5 × $10^{4}$ HH cells and Hut78 cells with 200 μ L FBS-free 1640 were seeded into the upper chamber of the well, respectively. The wells were inserted into the lower chambers with 600 μ L 1640 and 20% FBS. A total of 5 × $10^{4}$ MJ cells with 200 μ L FBS-free IMDM were seeded into the upper chamber of the well. The wells were inserted into the lower chambers with 600 μ L of IMDM and 40% of FBS. In terms of invasion, the upper chamber was first coated with Matrigel. The membrane of the well was fixed with methanol and subsequently stained with crystal violet after 16 h of incubation. After staining, the wells were washed with water and photos were taken of the cells on the membrane under a microscope.

### 4.11. RNA High-Throughput Sequencing (RNA-Seq)

RNAs were extracted from the shNC and shMETTL3 of HH cells. RNA sequencing libraries were subsequently generated using poly(A) RNA purified with a PolyT tract mRNA isolation system. Generated libraries were then sequenced on the Illumina high-throughput sequencing platform (NovaSeq 6000, Illumina, CA, USA), according to the manufacturer’s recommendations.

### 4.12. Gene Ontology and KEGG Pathway Analysis

To investigate biological processes, cellular components, and molecular functions associated with differentially expressed mRNAs and proteins, GO and KEGG pathway analyses were performed. Differential expression associated with GO and KEGG terms was assessed using Fisher’s Exact Test.

### 4.13. RNA Stability Assay

Actinomycin D (10 mg/mL) was applied to HH and MJ cells transduced with shNC or shMETTL3. RNA samples were collected at 0, 3, and 6 h, and ARHGEF12 and METTL3 mRNA levels were assessed by RT-qPCR.

### 4.14. Nude Mouse Xenograft Study

Six-week-old male NOD-Prkdcem26Cd52Il2rgem26Cd22/Nju NCG nude mice were subcutaneously injected with 5 × $10^{6}$ HH cells. Tumor volume was measured bi-daily, and tumor volume was calculated as (length × ${\mathrm{width}}^{2}$ )/2. Mice were sacrificed after 16 days, and tumors were collected and weighed. This protocol was approved by our institutional animal care committee.

## 5. Conclusions

Collectively, our work revealed the regulatory role of METTL3 in the progression and metastasis of CTCL cells both in vitro and in vivo. This function is related to ARHGEF12, a potential “executor” of METTL3. Mechanistically, METTL3 recognizes the m6A sites on ARHGEF12 and promotes its protein expression by affecting its mRNA stability. METTL3 and its downstream ARHGEF12 play a critical role in CTCL proliferation and metastasis and could serve as a potential therapeutic target for interventions.

## Data Availability

The datasets in study can be found in NCBI GEO, http://www.ncbi.nlm.nih.gov/bioproject/1243559 (accessed on 1 January 2025).

## Abbreviations

The following abbreviations are used in this manuscript:

CTCL	Cutaneous T-cell lymphoma
m6A	N6-methyladenosine
MF	Mycosis fungoides
pcALCL	Primary cutaneous anaplastic large-cell lymphoma
TBST	Tris-buffered saline with Tween 20
