# Correction: Gan et al. METTL3 Promotes Cutaneous T-Cell Lymphoma Progression by Regulating ARHGEF12 Expression. *Int. J. Mol. Sci.* 2025, *26*, 3640

**DOI:** 10.3390/ijms262311692

**Published:** 2025-12-03

**Authors:** Lu Gan, Yingqi Kong, Haoze Shi, Congcong Zhang, Cuicui Tian, Hao Chen

**Affiliations:** Hospital for Skin Diseases, Institute of Dermatology, Chinese Academy of Medical Sciences and Peking Union Medical College, Nanjing 210042, China

## 1. Errors in Figures

In the original publication [1], there were mistakes in Figures 3 and 6 as published. Figure 3, flow cytometry panels: incorrect images were displayed due to a layout error during figure assembly. Figure 3, transwell assay analysis: statistical inaccuracies occurred because the same dataset was inadvertently selected twice during graph generation, leading to erroneous quantitative representation. Figure 6, Western blot analysis: statistical inaccuracies occurred because the wrong dataset was inadvertently selected during graph generation, resulting in erroneous quantitative representation.

The corrected Figure 3 appears below.

**Figure 3:**
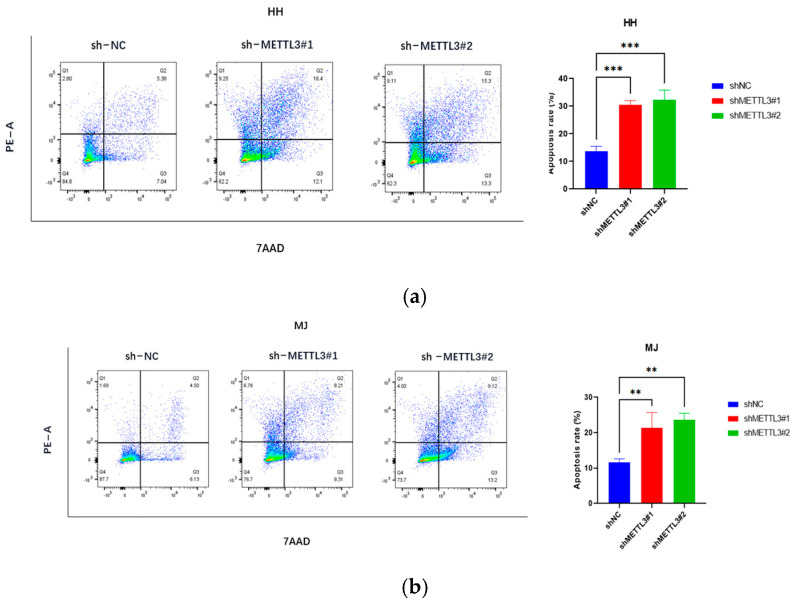

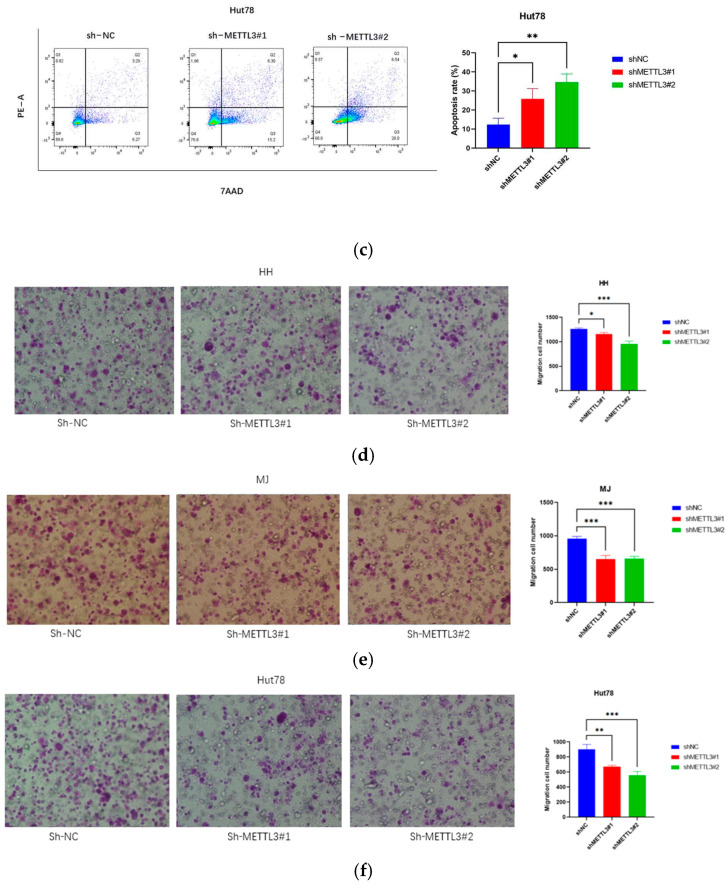
METTL3 downregulation inhibits CTCL cell invasion and promotes apoptosis: (**a**–**c**) Apoptosis assays showed significantly increased apoptosis rates in CTCL cells following METTL3 knockdown. (**d**–**f**) Transwell experiments indicated that METTL3 downregulation significantly impaired the migration ability of HH, MJ, and Hut78 cells within 16 h. All data are presented as mean ± S.D. and statistical significance is denoted as * *p* < 0.05, ** *p* < 0.01, and *** *p* < 0.001.

The corrected Figure 6 is provided below.

**Figure 6:**
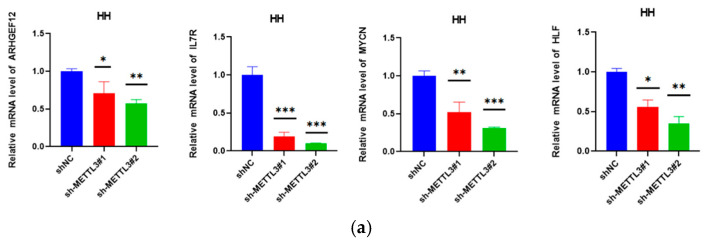

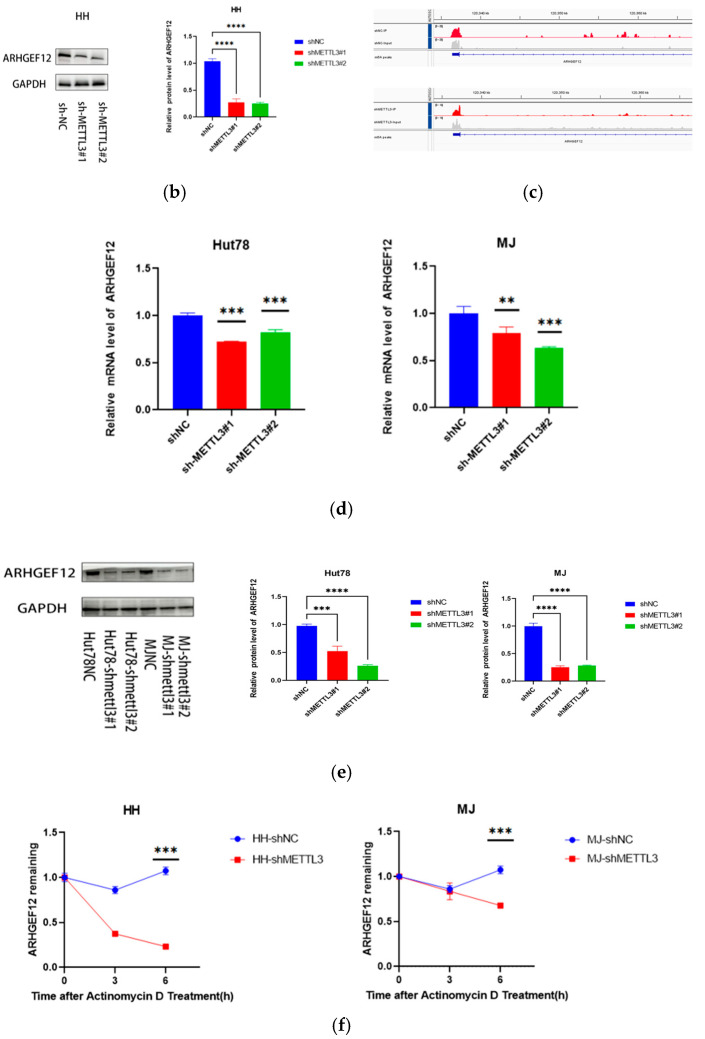
METTL3 affected ARHGEF12 mRNA stability: (**a**) MYCN, IL7R, ARHGEF12, and HLF mRNA levels in shNC and shMETTL3 cells in HH. (**b**) ARHGEF12 protein level in shNC and shMETTL3 cells in HH cells. (**c**) M6A peak in the 3′ UTR of ARHGEF12 in both shNC and shMETTL3 HH cells. (**d**,**e**) ARHGEF12 mRNA and protein levels in shNC and shMETTL3 cells in MJ and Hut78 cell lines. (**f**) RT-qPCR analysis of ARHGEF12 after actinomycin D treatment in shMETTL3 or control HH and MJ cells. All data are presented as the mean ± S.D. and statistical significance is denoted as * *p* < 0.05, ** *p* < 0.01, *** *p* < 0.001, and **** *p* < 0.0001.

## 2. Text Corrections

Errors have been identified in the original publication [1]. One sentence did not include the appropriate citation. Additionally, the nucleotide sequences provided for the lentiviral constructs contained misaligned duplications due to accidental text displacement during manuscript preparation.

A correction has been made to the Introduction section, third paragraph:

Histone deacetylases (HDACs) are ubiquitously expressed enzymes that catalyze the removal of acetyl groups from histones, leading to chromatin condensation and transcriptional repression. HDAC inhibitors (HDACis) induce tumor cell cycle arrest and apoptosis by inhibiting specific HDAC isoforms, thereby increasing histone acetylation and promoting chromatin remodeling. Representative HDACis include cedarbenamide, vorinostat, and romidepsin. A multicenter prospective trial reported an overall response rate of 39.06% for cedarbenamide monotherapy in refractory peripheral T-cell lymphoma, which increased to 51.18% when combined with chemotherapy [6]. Brentuximab vedotin (BV), an anti-CD30 antibody–drug conjugate, has been approved by the U.S. Food and Drug Administration (FDA) and the European Medicines Agency (EMA) for the treatment of CD30-positive mycosis fungoides (MF). BV is also indicated for relapsed or refractory classical Hodgkin lymphoma. CD30 expression is widely observed in lymphomatoid papulosis (LyP) and primary cutaneous anaplastic large-cell lymphoma (pcALCL). In MF and Sézary syndrome (SS), the proportion of CD30-positive tumor cells ranges from approximately 12% to 23%, increasing to 48% to 55% following large-cell transformation in MF patients. In an 18-month follow-up study conducted by Muniesa involving 67 patients (48 with MF, 7 with SS, and 12 with CD30+ lymphoproliferative disorders [LPD]), BV achieved an overall response rate (ORR) of 67% (63% in MF, 71% in SS, and 84% in CD30+ LPD). The median time to response was 2.8 months. During follow-up, 54% of patients (*n* = 36) experienced disease recurrence or progression in the skin, with a median progression-free survival (PFS) of 10.3 months [7]. Mogamulizumab, a humanized monoclonal antibody targeting CC chemokine receptor 4 (CCR4), was evaluated in a Japanese cohort of 28 patients with CTCL, demonstrating an ORR of 39.3% [8]. A phase III trial comparing mogamulizumab with vorinostat in refractory MF/SS reported a median PFS of 7.7 months with mogamulizumab, significantly longer than the 3.1 months observed in the vorinostat group [9].

An error was identified in the section Materials and Methods, Section 4.6. Plasmid Construction and Lentiviral Transfection:

To construct the lentiviral vector carrying shRNA targeting METTL3, complementary sense and antisense oligonucleotides were synthesized and subsequently cloned into the GV112 vector (hU6-MCS-CMV-puromycin). The sequence of sgNC was TTCTCCGAAC-GTGTCACGT. The shMETTL3 sequences designed to target METTL3 were as follows: ACCCACCTCTGGTGGCCCTAA; GTGCAGAACAGGACTCGACTA; and AGGCTCAACATACCCGTACTA. All lentiviral vectors and plasmids were generated by GeneChem (Shanghai, China). HH, Hut78, and MJ cells were transduced with lentiviral supernatants carrying shMETTL3 and were selected with 2 μg/mL puromycin for 48 h post-transfection.

## 3. References Corrections

The following reference is missing in the original publication [1]. With this correction, the order of some references has been adjusted accordingly.

9. Kim, Y.H.; Bagot, M.; Pinter-Brown, L.; Rook, A.H.; Porcu, P.; Horwitz, S.M.; Whittaker, S.; Tokura, Y.; Vermeer, M.; Zinzani, P.L. Mogamulizumab versus vorinostat in previously treated cutaneous T-cell lymphoma (MAVORIC): An international, open-label, randomised, controlled phase 3 trial. *Lancet Oncol.* **2018**, *19*, 1192–1204.

The authors state that the scientific conclusions are unaffected. This correction was approved by the Academic Editor. The original publication has also been updated.
